# Complete Androgen Insensitivity Syndrome: From the Relevance of an Accurate Genetic Diagnosis to the Challenge of Clinical Management. A Case Report

**DOI:** 10.3390/medicina57111142

**Published:** 2021-10-21

**Authors:** Federica Barbagallo, Rossella Cannarella, Matteo Bertelli, Andrea Crafa, Sandro La Vignera, Rosita A. Condorelli, Aldo E. Calogero

**Affiliations:** 1 Department of Clinical and Experimental Medicine, University of Catania, 95123 Catania, Italy; federica.barbagallo11@gmail.com (F.B.); crafa.andrea@outlook.it (A.C.); sandrolavignera@unict.it (S.L.V.); rosita.condorelli@unict.it (R.A.C.); aldo.calogero@unict.it (A.E.C.); 2 MAGI EUREGIO, 39100 Bolzano, Italy; matteo.bertelli@assomagi.org

**Keywords:** complete androgen insensitivity syndrome, disorders of sexual development, androgen receptor, *AR* gene

## Abstract

*Introduction*: Androgen insensitivity syndrome (AIS), an X-linked recessive disorder of sex development (DSD), is caused by variants of the androgen receptor (*AR*) gene, mapping in the long arm of the X chromosome, which cause a complete loss of function of the receptor. *Case presentation*: We report a patient diagnosed with complete AIS (CAIS) at birth due to swelling in the bilateral inguinal region. Transabdominal ultrasound revealed the absence of the uterus and ovaries and the presence of bilateral testes in the inguinal region. The karyotype was 46,XY. She underwent bilateral orchiectomy at 9 months and was given estrogen substitutive therapy at the age of 11 years. Genetic analysis of the AR gene variants was requested when, at the age of 20, the patient came to our observation. *Methods*: The genetic testing was performed by next-generation sequence (NGS) analysis. *Results*: The genetic analysis showed the presence of the c.2242T>A, p.(Phe748Ile) variant in the *AR* gene. To the best of our knowledge, this variant has not been published so far. Furthermore, the patient has a heterozygous c.317A>G, p.(Gln106Arg) variation of the gonadotropin-releasing hormone receptor (*GNRHR*) gene, a heterozygous c.2273G>A, p.Arg758His variation of the chromodomain helicase DNA binding protein *7* (*CHD7*) gene, and compound heterozygous c.875A>G, p.Tyr292Cys, and c.8023A>G, p.Ile2675Val variations of the Dynein Axonemal Heavy Chain *11* (*DNAH11*) gene. *Conclusions*: The case herein reported underlines the importance of an accurate genetic analysis that has to include karyotype and *AR* gene variant analysis. This is useful to confirm a clinical diagnosis and establish the proper management of patients with CAIS. Numerous variants of the *AR* gene have not yet been identified. Moreover, several pitfalls are still present in the management of these patients. More studies are needed to answer unresolved questions, and common protocols are required for the clinical follow-up of patients with CAIS.

## 1. Introduction

Androgen insensitivity syndrome (AIS) is an X-linked recessive disorder of sex development (DSD). It is caused by several variants of the androgen receptor (*AR*) gene that maps in the long arm of the X chromosome [1]. According to the severity of AR resistance to androgens, AIS is classified as complete AIS (CAIS), partial AIS (PAIS), and mild AIS (MAIS).

CAIS is characterized by complete resistance to the effects of androgens. It was first described by Morris in 1953 [2]. CAIS is still considered a rare disorder, with a frequency ranging from 1:20,400 to 1:99,100 male births [3].

Patients with CAIS have a female phenotype with a 46,XY karyotype. CAIS is characterized by testes located in the abdomen, inguinal ring, or labio-scrotal region, female external genitalia, absence of uterus and ovaries, and a blind-ending vagina [1]. In patients with intact gonads, puberty occurs spontaneously with normal breast development and female body adiposity due to peripheral aromatization of testosterone [4]. 

Currently, approximately 1000 variants in the *AR* gene have been associated with AIS [5]. The AR is encoded by a gene mapping in the Xq11-12 chromosome that consists of eight exons [6]. The AR protein has four functional domains: the N-terminal domain (NTD, exon 1), the DNA-binding domain (DBD, exons 2 and 3), the hinge region, and the ligand- or androgen-binding domain (LBD, exons 4–8) [7]. Upon binding of androgens to the LBD, the ligand–receptor complex translocates into the nucleus, dimerizes, and after the interaction of DBD with androgen-responsive elements (ARE), activates the transcription of androgen-responsive genes [8]. 

The majority of the *AR* variants have been discovered in the LBD region that can alter several functions of the receptor, such as its ligand-binding ability and the interplay with other coactivators [1]. In about two-thirds of the cases, variants in the *AR* gene originate from germ cells of asymptomatic mothers, whereas in other cases, they originate in somatic cells or are de novo variants [9]. 

We herein describe the case of a patient with CAIS who showed a missense variant of the *AR* gene that, to the best of our knowledge, has never been published.

## 2. Case Presentation

At the age of 20 years, the patient came to our observation with the diagnosis of CAIS. Her past medical history revealed bilateral swelling in the inguinal region at birth. She had female external genitalia. Genital exams showed slightly hypertrophic labia majora, and normal labia minora, clitoris, and urethral meatus, and the vaginal opening was normally located. Transabdominal ultrasound revealed the absence of uterus and ovaries and the presence of bilateral testes in the inguinal region, at the level of the internal inguinal ring. Chromosome analysis was performed and showed a 46,XY karyotype. The laparoscopy confirmed the results described by ultrasound. At nine months, the patient underwent bilateral orchiectomy with the removal of the undescended testes for the increased risk of malignancy. The histological examination of the removed gonads showed two hypotrophic testes with seminiferous tubules consisting mainly of Sertoli cells and few spermatogonia, associated with Leydig cell hyperplasia. The epididymis was also fibrotic and hypotrophic. All these findings were consistent with CAIS. At the age of 11 years, the patient was prescribed hormone replacement therapy (HRT) with oral ethinylestradiol for the induction of puberty, with a gradual increment of the dosage. 

The patient was referred to our Division at the age of 20 years. At the moment of our first visit, she was under treatment with 17ß-estradiol transdermal patch at the dose of 25 µg/day. At general physical examination, she had well-represented adipose tissue, normally developed breasts (V Tanner stage), and scanty axillary and pubic hair. The patient weighed 83.4 kg (kg) and was 1.675 m (m) tall; her body mass index (BMI) was 29.8 kg/m^2^. Skin hyperpigmentation was present in both axillae, suggesting the presence of acanthosis nigricans. Vaginal examination revealed a blind-ending vagina of a normal dimension. Hormonal levels showed elevated serum FSH (81.86 UI/L, female normal values in the follicular phase 3.03–8.08 IU/L), LH (20.78 IU/L, female normal values in the follicular phase 21–251 IU/L), 17ß-estradiol (35 ng/mL, female normal values in the follicular phase 21–251 ng/mL) in the lower limit of the female range, and testosterone levels in the normal female range (0.25 ng/mL, normal values 0.07–0.79). The lipid profile was within the normal limit, whereas insulin resistance was found (HOMA index = 3.22). Bone mineral density (BMD) assessed by Dual-Energy X-ray Absorptiometry (DEXA) showed osteopenia at the lumbar spine (T-score = −2.3; BMD 0.950 g/centimeters^2^). 

We performed genetic testing to evaluate *AR* gene variants by Next-Generation Sequence (NGS) analysis. The results showed a novel missense variant of the *AR* gene that confirmed the clinical diagnosis of CAIS. 

The dose of transdermal 17ß-estradiol was increased to 30 µg to achieve hormonal values compensation able to maintain female secondary sexual characters, prevent bone loss and neurocognitive disorders, and guarantee cardiovascular health and psychophysical well-being. Calcium and vitamin D supplementation was started to prevent osteoporosis. A hypoglycemic low-calorie diet and regular physical activity were prescribed. 

## 3. Materials and Methods

The genetic testing was performed by NGS analysis. The DNA of the patient was extracted from peripheral blood using a commercial kit (SAMAG BLOOD DNA Extraction Kit). The proband’s DNA was used for NGS on a MiSeq Illumina instrument. The target regions were enriched by the Illumina Nextera Rapid Capture Enrichment kit. 

The patient was tested for the pre-determined gene panel for female infertility, a miscellaneous panel that included DSD (Table 1). 

The variants were filtered as follows: (1) variants with minor allele frequency (MAF) of less than 1% in 1000 Genomes (http://www.1000genomes.org/home, accessed on 1 September 2021) [EVS (http://evs.gs.washington.edu/EVS/, accessed on 1 September 2021) and GNOMAD (https://gnomad.broadinstitute.org/, accessed on 1 September 2021) databases were considered]; (2) evaluation was focused on coding exons along with flanking +/−15 intronic bases; (3) for synonymous and splicing variants with GMAF/MAX MAF clearly inferior to the known frequency of the pathology, the presence in the database HGMD (The Human Gene Mutation Database) was verified. The interpretation of variants was carried out according to the scoring system by the American College of Medical Genetics and Genomics (ACMG) guidelines. All variants related to the phenotype of the patient, except benign or likely benign variants, are reported. The highlighted variants were classified into pathogenetic, probably pathogenic, and of uncertain significance. Bioinformatics tools were used to predict pathogenicity in silico (such as SIFT, MutationTaster, PROVEAN, Polyphen2) and to evaluate the evolutionary conservation for missense variants.

## 4. Results

We identified the following missense variants: (1) c.2242T>A in the *AR* gene that causes the p.(Phe748IIe) amino acid variation; (2) the heterozygous c.317A>G, p.(Gln106Arg) variation in the gonadotropin-releasing hormone receptor (*GNRHR*) gene; (3) the heterozygous c.2273G>A, p.Arg758His variation in the chromodomain helicase DNA binding protein 7 (*CHD7*) gene; (4) the c.875A>G, p.Tyr292Cys and the c.8023A>G, p.Ile2675Val compound heterozygous variations in the Dynein Axonemal Heavy Chain 11 (*DNAH11*) gene (Table 2).

The *AR* c.2242T>A variation is not listed in the ClinVar database and has never been described before. Furthermore, the variant p.(Phe748IIe) is in an amino acid residue conserved during evolution. The amino acid 748 is located in the LBD region. The variant c.2242T>A p.(Phe748Ile) is a missense of pathogenic significance because it alters the function of the LBD region that is responsible for the binding of androgen to AR. Mutations in the LBD region of the *AR* gene have already been reported to be associated with CAIS. This variant was predicted to be disease-causing, deleterious, and probably damaging by the databases ClinVar, LOVD, HGMD.

The *GNRHR* c.317A>G, p.(Gln106Arg) is a missense variant with low allelic frequency (gnomAD MAX_MAF: 0.284%) of pathogenic significance, as predicted by the databases ClinVar, LOVD, HGMD. However, heterozygosis and the serum levels of LH and FSH excluded its role in the patient’s phenotype. 

The *CHD7* c.2273G>A, p.Arg758His variation is a missense one with low allelic frequency (gnomAD MAX_MAF: 0.00724%). Although predicted as pathogenic or likely pathogenic by the databases ClinVar, LOVD, HGMD, it has been described in healthy subjects, suggesting the possible benignity of this variant. 

The *DNAH11* c.875A>G, p.(Tyr292Cys) variation is a missense one with low allelic frequency (gnomAD MAX_MAF: 0.00185%), predicted as pathogenic or likely pathogenic by the databases ClinVar, LOVD, HGMD. The heterozygosis of this variation actually excluded its role in the patient’s phenotype. The *DNAH11* c.8023A>G, p.Ile2675Val is a missense variant with low allelic frequency (gnomAD MAX_MAF: 0.176%) predicted as pathogenic or likely pathogenic by the databases ClinVar, LOVD, HGMD.

## 5. Discussion

CAIS has several different clinical presentations depending on the age of the patient. CAIS should be suspected in a female neonate with an inguinal hernia or with swelling of the labia majora [1]. In fact, an inguinal hernia is a rare condition in the pediatric population (1–4%) with a clear prevalence in boys (10:1). Thus, karyotype analysis should be requested for all female children with mono- or bilateral inguinal hernia [10]. However, CAIS is often diagnosed at puberty when the patient presents with primary amenorrhea, owing to the absence of the uterus [1]. Nowadays, the karyotype of a fetus is frequently known before birth because different analyses, such as chorionic villi analysis, amniocentesis, maternal circulating free fetal DNA analysis, are more frequently performed. Thus, CAIS can also be diagnosed because of a mismatch between prenatal sex prediction and the phenotype at fetal ultrasound scans or birth [11]. Diagnosis may also result from a known family history of CAIS [11]. 

The female phenotype with a 46,XY karyotype is a complex clinical condition, and its diagnosis can sometimes be difficult. Although CAIS is the most common cause for the 46,XY DSD, it is not the only one. The 46,XY DSD consists of several disorders with different genetic backgrounds [12]. Wrong or inaccurate diagnosis can be risky because it precludes proper clinical management. In the past, the diagnosis was based mainly on clinical signs. However, molecular and genetic testing is indispensable to identify the correct etiology [13]. Minto and colleagues [14] reviewed the diagnosis of 46 adult patients with female phenotype and 46,XY karyotype. They found that 13% of them received an incorrect diagnosis. In another study, Costagliola and colleagues found about 10% of misdiagnoses in a group of women with 46,XY karyotype who had previously received a diagnosis of CAIS [12]. Therefore, the authors suggested a re-evaluation of old diagnoses in light of the new diagnostic techniques. A proper diagnosis is essential to enable appropriate management and genetic counseling of these patients and their families [12]. The patient we described received a molecular diagnosis only when she was 20 years old, despite her being diagnosed with CAIS at birth. The variation c.2242T>A p.(Phe748Ile) of the *AR* gene was found, and, to the best of our knowledge, this variant has never been described before. It is located in exon 5 of the *AR* gene. It is located in the LBD region of the *AR* gene, which is essential for the binding of androgens. The LBD region (amino acids 646–920) is encoded by exons 4–8 and contains specific ligand-binding sites for androgens, various transcription factors of co-activation, and the activation function-2 (AF-2) region [15]. In basal condition, AR is in the cytoplasm where it forms a multimeric complex with heat shock proteins (HSPs). Then, the LBD region allows the interaction with androgens and, in turn, AR migration into the nucleus [1]. 

Currently, more than 1000 different variants have been associated with AIS [5]. Many of them involve the LBD region [15]. The most common functional defect of the *AR* gene is due to alterations of the hydrophobic ligand-binding pocket, which is fundamental for the repositioning of helix 12 to form the AF-2 co-regulator binding surface [11]. The largest number of variants of the LBD region that cause CAIS are located between amino acid residues 688 and 712, 739 and 784, and 827 and 870 [11]. Specifically, the first two clusters represent most of the ligand-binding pocket. The variation identified in our patient involves the amino acid 748. Thus, variants in the LBD or DBD regions seem to impair AR function more severely than variants in other regions of the receptor and, therefore, are more frequently associated with CAIS or PAIS [16]. Conversely, variants in the N-terminal region seem to be more frequently associated with MAIS and have been more commonly reported in patients with isolated male infertility [16]. Thus, *AR* gene variants may play a role in the genetic cause of male infertility [16].

The diagnosis and management of CAIS represent a difficult challenge for clinicians. Many questions are unresolved for the best treatment of these patients. First of all, the correct timing of orchiectomy is still debated. Traditionally, gonadectomy has historically been suggested to avoid the malignant transformation of undescended testes. However, more recent studies recommend delaying gonadectomy until after puberty [4,13,17]. In fact, the risk of malignancy is very low in children and increases with age [4]. Thus, the maintenance of gonads until puberty may optimize the growth and acquisition of bone mass in patients with CAIS [4]. Moreover, postponed gonadectomy also allows patient’s independent decision about surgery once adulthood is reached. In addition, the psychological impact of gonadectomy should be considered [4]. In a study conducted on 14 women with CAIS (mean age 48 years), 80% of the patients considered the most appropriate timing for gonadectomy or vaginoplasty during adolescence or adulthood [18]. Certainly, a proper follow-up of the undescended retained testes should be performed in patients with CAIS when gonadectomy is postponed [1]. 

Another important question regards hormonal replacement therapy (HRT), which is fundamental in patients with CAIS after bilateral gonadectomy. The classic HRT for patients with CAIS is based on estrogen administration, although there are no clear guidelines on hormone formulation, dosages, and monitoring [9]. Despite good compliance to HRT, many patients with CAIS report decreased well-being and sexual satisfaction [19,20]. An improvement in sexual health has been reported in CAIS patients treated with testosterone [20]. However, more studies are needed to evaluate the efficacy of treatment with testosterone in patients with CAIS. 

Bone health and metabolic aspects are two other important clinical issues in the management of patients with CAIS. Patients with CAIS but without gonads have reduced BMD on DXA and an increased risk of osteoporosis [21]. Numerous mechanisms are responsible for the impaired bone health in patients with CAIS, such as loss of AR signaling at the bone level, gonadectomy, age at gonadectomy (before or after the achievement of the peak bone mass), inadequate HRT, poor compliance with therapy, high serum FSH levels, lack of INSL-3 after orchiectomy [22]. Moreover, it has been shown that vitamin D receptor (VDR) and the microsomal enzyme cytochrome P450, subfamily 2R, polypeptide 1 (CYP2R1), responsible for vitamin D hydroxylation in position 25, are expressed in the testis [23]. Experimental and clinical studies suggest that the testis produces 25-hydroxy-vitamin D3 [22]. Thus, gonadectomy may impair bone health in patients with CAIS not only for the lack of androgens but also through other mechanisms such as the loss of INSL-3 and vitamin D activation. A proper HRT may improve bone health, although it seems unable to normalize BMD [21]. Our patient presented with osteopenia at the lumbar spine (T-score = −2.3; BMD 0.950 g/centimeters^2^) despite her young age. She also had a BMI of 29.8 kg/m^2^ and insulin resistance. Alterations of the AR receptor can be associated with an increased risk of metabolic syndrome, diabetes, and cardiovascular disease. Studies conducted on AR knockout (ARKO) mice have shown that they become obese without any changes in food intake and that hyperinsulinemia, hyperglycemia, hypertriglyceridemia, and impaired glucose tolerance developed with aging [24]. Further studies have to evaluate the metabolic consequences in women with CAIS.

## 6. Proposal for the Clinical Management of Patients with CAIS

This case report underlines the complexity of the management of CAIS and the urgent need to standardize the diagnostic and therapeutic strategies for these patients. Once CAIS is suspected in female prepubertal children with bilateral inguinal/labial hernia or female adolescents with primary amenorrhea, the diagnosis needs to be confirmed. Hormonal assessment could be helpful in suggesting the clinical diagnosis, and image diagnosis would show the absence of Mullerian structures and the presence of testes [1]. Then, a karyotype exam should be performed to identify female patients 46,XY. Finally, *AR* gene analysis is mandatory to confirm a definitive diagnosis of CAIS [1]. Current evidence suggests delaying gonadectomy until at least puberty [4,13,17]. Thus, a proper follow-up of the retained testes should be performed in these patients. After surgical removal of the gonads, HRT is mandatory to avoid symptoms of hypoestrogenism. The objective of HRT depends on the time of gonadectomy. As previously discussed, there is not a unique HRT protocol for CAIS patients [9]. Both oral and transdermal estradiol formulations seem to be effective, even if the latter could be more physiological and safer due to the lower first-pass effect of liver metabolism, lower interference with IGF-1 serum levels, and reduced risk of thromboembolism [25]. Assessment of bone health is essential, and the supplementation with calcium and vitamin D should be considered in patients with reduced BMD. Bisphosphonate therapy may be considered in patients with severe osteoporosis and/or fractures [5]. Furthermore, because of the high risk of metabolic disorders, clinicians should evaluate body composition, glucose and lipid metabolism, and cardiovascular risk in patients with CAIS [1]. Finally, the psychological aspect of patients with CAIS should not be undervalued. Psychological support is an essential component of the correct management of these patients [26]. 

## 7. Conclusions

The 46,XY DSD individuals are a group of patients with a complex clinical condition. It consists of several diseases that require accurate differential diagnoses for the correct management of these patients. CAIS is the most common disease of the 46,XY DSD group. The case herein reported underlines the importance of genetic testing that should comprehend the analysis not only of the karyotype but also of *AR* gene variants. The latter is mandatory to confirm a clinical diagnosis and to establish the best hormonal, surgical (if necessary), and psychological management. Since numerous variants of the *AR* gene have not yet been identified, a re-evaluation of old diagnoses of CAIS should be performed in the light of the new diagnostic genetic techniques, among which NGS. Several drawbacks are still present in the management of these patients. More studies are needed to answer the unresolved questions about the timing of gonadectomy, the follow-up of patients with gonads, proper HRT, bone health, and metabolic consequences. In clinical practice, CAIS requires a multidisciplinary team and a close collaboration between endocrinologists, gynecologists, surgeons, and psychiatrists.

## Figures and Tables

**Table 1:** Genes included in the panel of female infertility.

**Genes** **(OMIM Number)**	*DGRG2* (OMIM: 300572), *AR* (OMIM: 313700), *AXL* (OMIM: 109135), *CATSPER1* (OMIM: 606389), *CCDC141* (OMIM: 616031), *CCDC40* (OMIM: 613799), *CCNO* (OMIM: 607752), *CFAP20DC* (OMIM: 300572), *CFAP43* (OMIM: 617558), *CFAP44* (OMIM: 617559), *DNAAF1* (OMIM: 613190), *DNAAF3* (OMIM: 614566), *DNAAF4* (OMIM: 608706), *DNAAF6* (OMIM: 300933), *DNAH11* (OMIM: 603339), *DNAH8* (OMIM: 603337), *DNAI2* (OMIM: 605483), *DNAL1* (OMIM: 610062), *DRC1* (OMIM: 615288), *FGF17* (OMIM: 603725), *FGFR1* (OMIM: 136350), *FSHB* (OMIM: 136530), *GLI2* (OMIM: 165230), *GNRHR* (OMIM: 138850), *HS6ST1* (OMIM: 604846), *HYDIN* (OMIM: 610812), *KISS1* (OMIM: 603286), *KLHL10* (OMIM: 608778), *LHX3* (OMIM: 600577), *LRRC6* (OMIM: 614930), *MEIOB* (OMIM: 617670), *NANOS1* (OMIM: 608226), *NR5A1* (OMIM: 184757), *OTX2* (OMIM: 600037), *PLCZ1* (OMIM: 608075), *POU1F1* (OMIM: 173110), *PROK2* (OMIM: 607002), *PROP1* (OMIM: 601538), *RSPH3* (OMIM: 615876), *RSPH9* (OMIM: 612648), *SEMA3E* (OMIM: 608166), *SLC26A8* (OMIM: 608480), *SOX10* (OMIM: 602229), *SPAG1* (OMIM: 603395), *SPRY4* (OMIM: 607984), *SRA1* (OMIM: 603819), *SUN5* (OMIM: 613942), *SYCP3* (OMIM: 604759), *TACR3* (OMIM: 162332), *TEX11* (OMIM: 300311), *USP9Y* (OMIM: 400005), *ZMYND10* (OMIM: 607070), *ZPBP* (OMIM: 608498), *ANOS1* (OMIM: 300836), *AURKC* (OMIM: 603495), *BRDT* (OMIM: 602144), *CCDC103* (OMIM: 614677), *CCDC39* (OMIM: 613798), *CCDC65* (OMIM: 611088), *CENPF* (OMIM: 600236), *CFAP298* (OMIM: 615494), *CFAP44* (OMIM: 617559), *CHD7* (OMIM: 608892), *DNAAF2* (OMIM: 612517), *DNAAF3* (OMIM: 614566), *DNAAF5* (OMIM: 614864), *DNAH1* (OMIM: 603332), *DNAH5* (OMIM: 603335), *DNAI1* (OMIM: 604366), *DNAJB13* (OMIM: 610263), *DPY19L2* (OMIM: 613893), *DUSP6* (OMIM: 602748), *FGF8* (OMIM: 600483), *FLRT3* (OMIM: 604808), *GAS8* (OMIM: 605178), *GNRH1* (OMIM: 152760), *HESX1* (OMIM: 601802), *HSF2* (OMIM: 140581), *IL17RD* (OMIM: 606807), *KISS1R* (OMIM: 604161), *LHB* (OMIM: 152780), *LHX4* (OMIM: 602146), *MCIDAS* (OMIM: 614086), *MEIOB* (OMIM: 617670), *NME8* (OMIM: 607421), *NSMF* (OMIM: 608137), *PICK1* (OMIM: 605926), *PLK4* (OMIM: 605031), *PROK2* (OMIM: 607002), *PROKR2* (OMIM: 607123), *RSPH1* (OMIM: 609314), *RSPH4A* (OMIM: 612647), *SEMA3A* (OMIM: 603961), *SEPTIN12* (OMIM: 611562), *SOHLH1* (OMIM: 610224), *SOX3* (OMIM: 313430), *SPATA16* (OMIM: 609856), *SPRY4* (OMIM: 607984), *STK36* (OMIM: 607652), *SYCE1* (OMIM: 611486), *TAC3* (OMIM: 162330), *TAF4B* (OMIM: 601689), *TEX15* (OMIM: 605795), *WDR11* (OMIM: 606417), *ZMYND15* (OMIM: 614312).

**Table 2:** Gene variations found in the patient.

Gene	Exon	Type	dbSNP	Nucleotide	Amino acid	Zygosity	Clinical Relevance	Inheritance
*AR*	ex5	Missense	---	NM_000044.6 c.2242T>A	NP_000035.2 p.(Phe748Ile)	hemizygosity	Pathogenetic	XLR
*GNRHR*	ex1	Missense	rs104893836	NM_000406.3 c.317A>G	NP_000397.1 p.(Gln106Arg)	heterozygosity	Pathogenetic	AR
*CHD7*	ex5	Missense	rs202208393	NM_017780.4 c.2273G>A	NP_060250.2 p.(Arg758His)	heterozygosity	VUS	AD
*DNAH11*	ex49	Missense	rs72657364	NM_001277115.2 c.8023A>G	NP_001264044.1 p.(Ile2675Val)	heterozygosity	Likely benign	AR
*DNAH11*	ex4	Missense	rs768404895	NM_001277115.2 c.875A>G	NP_001264044.1 p.(Tyr292Cys)	heterozygosity	VUS	AR

**Abbreviations:** AD, autosomal dominant; AR, autosomal recessive; *AR*, androgen receptor; *CHD7*, chromodomain helicase DNA binding protein 7; *DNAH11*, dynein axonemal heavy chain 11; *GNRHR*, gonadotropin releasing hormone receptor; VUS, variant of uncertain significance; XLR, X-linked recessive.

## Data Availability

Data are available upon request to the corresponding author.
